# 

**Published:** 1871-11

**Authors:** 


☞ Should any of our subscribers or exchanges fail to get THE COMPANION regularly, they will confer a favor by notifying us of the fact.

☞ We respectfully solicit Original Articles, Reports of Societies, Clinics, Home and Foreign Correspondence, News, etc., etc., of interest to medical readers.

☞ To insure publication, articles should be practical, as brief as possible, and carefully written, on one side of the paper. Articles should be received by the 15th of one month to insure publication in the next. ☞ Friends, send in your articles. ☜

### TO OUR SUBSCRIBERS.

It would be too troublesome and expensive to forward bills of subscription in every number of THE COMPANION—we therefore, earnestly ask all of our subscribers who have not paid their subscription, the very small sum of two dollars, to do so now, and you will receive your bill, receipted, in the next number. Friends, don't forget to promptly discharge this part of your duty towards enabling our publisher to furnish THE COMPANION monthly to you and your Professional brethren. Don't fail to comply at once with the above request.

